# PBOX-15, a novel microtubule targeting agent, induces apoptosis, upregulates death receptors, and potentiates TRAIL-mediated apoptosis in multiple myeloma cells

**DOI:** 10.1038/sj.bjc.6606035

**Published:** 2010-12-21

**Authors:** E N Maginn, P V Browne, P Hayden, E Vandenberghe, B MacDonagh, P Evans, M Goodyer, P Tewari, G Campiani, S Butini, D C Williams, D M Zisterer, M P Lawler, A M McElligott

**Affiliations:** 1 John Durkan Leukaemia Laboratories, Institute of Molecular Medicine, Trinity College Dublin, St James's Hospital, Dublin 8, Ireland; 2 Department of Haematology, St James's Hospital, Dublin, Ireland; 3 European Research Centre for Drug Discovery and Development, University of Siena, Siena, Italy; 4 School of Biochemistry and Immunology, Trinity College Dublin, Dublin, Ireland

**Keywords:** myeloma, caspase-8, DR5, TRAIL, bim

## Abstract

**Background:**

In recent years, much progress has been made in the treatment of multiple myeloma. However, a major limitation of existing chemotherapeutic drugs is the eventual emergence of resistance; hence, the development of novel agents with new mechanisms of action is pertinent. Here, we describe the activity and mechanism of action of pyrrolo-1,5-benzoxazepine-15 (PBOX-15), a novel microtubule-targeting agent, in multiple myeloma cells.

**Methods:**

The anti-myeloma activity of PBOX-15 was assessed using NCI-H929, KMS11, RPMI8226, and U266 cell lines, and primary myeloma cells. Cell cycle distribution, apoptosis, cytochrome *c* release, and mitochondrial inner membrane depolarisation were analysed by flow cytometry; gene expression analysis was carried out using TaqMan Low Density Arrays; and expression of caspase-8 and Bcl-2 family of proteins was assessed by western blot analysis.

**Results:**

Pyrrolo-1,5-benzoxazepine-15 induced apoptosis in *ex vivo* myeloma cells and in myeloma cell lines. Death receptor genes were upregulated in both NCI-H929 and U266 cell lines, which displayed the highest and lowest apoptotic responses, respectively, following treatment with PBOX-15. The largest increase was detected for the death receptor 5 (DR5) gene, and cotreatment of both cell lines with tumour necrosis factor-related apoptosis-inducing ligand (TRAIL), the DR5 ligand, potentiated the apoptotic response. In NCI-H929 cells, PBOX-15-induced apoptosis was shown to be caspase-8 dependent, with independent activation of extrinsic and intrinsic apoptotic pathways. A caspase-8-dependent decrease in expression of Bim_EL_ preceded downregulation of other Bcl-2 proteins (Bid, Bcl-2, Mcl-1) in PBOX-15-treated NCI-H929 cells.

**Conclusion:**

PBOX-15 induces apoptosis and potentiates TRAIL-induced cell death in multiple myeloma cells. Thus, PBOX-15 represents a promising agent, with a distinct mechanism of action, for the treatment of this malignancy.

Pyrrolo-1,5-benzoxazepine-15 (PBOX-15) is a novel tubulin depolymerising agent (Mulligan *et al*, 2006) that has been shown by us to exhibit proapoptotic activity in a variety of human tumour cell types, including those derived from both solid and haematological malignancies (Greene *et al*, 2008; McElligott *et al*, 2009; Nathwani *et al*, 2009; Bright *et al*, 2010). Recently, we have shown that PBOX-15 induces apoptosis in *ex vivo* B-cell chronic lymphocytic leukaemia (CLL) cells harbouring poor prognostic indicators and fludarabine resistance-associated p53 deletions (McElligott *et al*, 2009), and in imatinib-resistant chronic myeloid leukaemia (CML) cells (Bright *et al*, 2010). Importantly, PBOX-15 displays minimal toxicity towards normal blood and bone marrow cells (McElligott *et al*, 2009). The anticancer activity of drugs that interfere with tubulin dynamics, collectively known as microtubule targeting agents (MTA), is well established and, conventionally, the proapoptotic activity of these agents has been linked to their induction of mitotic arrest (Jordan and Wilson, 2004; Zelnak, 2007). However, it is becoming increasingly clear that additional mechanisms leading to cell death may also be activated by these agents (Gascoigne and Taylor, 2008), and indeed we have shown that PBOX-15 induces apoptosis independent of cell cycle arrest in *ex vivo* CLL cells (McElligott *et al*, 2009).

In this study, we investigate the efficacy and mode of action of PBOX-15 in multiple myeloma, a common B-cell malignancy. Myeloma is characterised by the accumulation of malignant plasma cells with defective apoptotic mechanisms and minimal proliferative rates (Kuehl and Bergsagel, 2005). Current chemotherapy options include the proteasome inhibitor bortezomib, thalidomide, or its immunomodulatory analogue lenalidomide, in combination with steroids and DNA alkylating agents (Kyle and Rajkumar, 2008). However, a major drawback of these agents is the eventual development of resistance. Of particular concern is the emergence of resistance to bortezomib and lenalidomide in myeloma patients (Politou *et al*, 2006; Schmidmaier *et al*, 2007; Sonneveld *et al*, 2008). Therefore, there is a pressing need for continued investigation and development of alternative treatment options for patients. The MTA vincristine has also demonstrated therapeutic efficacy in myeloma, and has previously been incorporated into initial treatment regimes for newly diagnosed patients (Alexanian *et al*, 1990). However, its use is associated with the development of multidrug resistance, and it has largely been replaced by newer agents (Grogan *et al*, 1993; Kyle and Rajkumar, 2008). A number of preclinical studies have demonstrated the anti-myeloma activity of other MTAs, including Taxol, Vinorelbine (a semisynthetic Vinca alkaloid), and the isocourmarin derivative 185322 (Aoyama *et al*, 1998; Ochiai *et al*, 2002; Kawano *et al*, 2007). In addition, 5HPP-33, a thalidomide analogue with potent anti-myeloma activity, has also demonstrated tubulin-polymerisation-inhibiting activity *in vitro* (Kizaki and Hashimoto, 2008).

In this study, we demonstrate the anti-myeloma activity of PBOX-15 in a panel of myeloma cell lines and in primary myeloma cells *ex vivo*. Moreover, we delineate the mechanism of PBOX-15 activity in myeloma cells: we show induction of caspase-8-dependent apoptosis, independent activation of the extrinsic and intrinsic apoptotic pathways, and early downregulation of the proapoptotic BH3-only protein Bim. Importantly, we show upregulation of death receptor 5 (DR5) in PBOX-15-treated myeloma cells, with resultant potentiation of apoptosis following cotreatment with PBOX-15 and the DR5 ligand, tumour necrosis factor-related apoptosis-inducing ligand (TRAIL).

## Materials and methods

### Chemicals

PBOX-15 was synthesised as previously described (Campiani *et al*, 1996; Mc Gee *et al*, 2005). *Killer*TRAIL was obtained from Alexis Biochemicals (Lausen, Switzerland) and caspase inhibitors were obtained from Calbiochem (Darmstadt, Germany). Unless indicated, all other reagents and chemicals were obtained from Sigma-Aldrich (St Louis, MO, USA).

### Cell culture

NCI-H929, U266, and RPMI8226 myeloma cell lines were obtained from the DSMZ cell bank (Braunschweig, Germany). KMS11 cells (Namba *et al*, 1989) were a kind gift from Dr Takemi Otsuki, Kawasaki Medical School, Japan. All cell lines were cultured in complete medium (RPMI-1640 medium supplemented with 10% fetal calf serum and 1% penicillin–streptomycin) under standard cell culture conditions.

### Patient samples

Written informed consent was obtained from five myeloma patients before sample collection, and the study was approved by the St James's Hospital and Federated Dublin Voluntary Hospitals’ Joint Ethics Committee, Dublin, Ireland. CD138^+^ cells were isolated from bone marrow aspirates on a MACS Separator using whole blood CD138 Microbeads (Miltenyi Biotec, Auburn, CA, USA), as per manufacturers’ instructions, or identified by gating of phycoerythrin (PE)-conjugated anti-CD138 (BD Biosciences, Franklin Lakes, NJ, USA)-stained cells on a CyAn ADP flow cytometer (Beckman Coulter, Brea, CA, USA) using Summit V4.3 software (Dako, Fort Collins, CO, USA).

### Cell cycle analysis and apoptosis assays

Cell cycle distribution was analysed by flow cytometry analysis of propidium iodide (PI; Invitrogen, Paisley, UK)-stained cells. Cells were fixed and permeabilised with 90% (v/v) ethanol, and incubated with 25 *μ*g ml^−1^ PI and 0.1 mg RNase A (Gentra Systems Inc., Minneapolis, MN, USA) for 30 min before analysis using a FACSCalibur flow cytometer and CellQuest software (BD Biosciences). Quantification of apoptosis was determined by flow cytometry analysis of cells costained with fluorescein isothiocyanate (FITC)-conjugated AnnexinV (AnnexinV-FITC) (IQ Products, Groningen, The Netherlands) and PI (AnnexinV/PI assay) as previously described (McElligott *et al*, 2009). For primary myeloma samples, cells were stained with PE-conjugated anti-CD138, AnnexinV-FITC, and Hoechst33258 at room temperature for 30 min, and analysed on a CyAn ADP flow cytometer. Mitochondrial inner membrane (MIM) depolarisation was assessed by incubating cells for 15 min with 2 *μ*M JC-1, a cationic dye, followed by flow cytometry analysis. Mitochondrial cytochrome *c* release was assessed using the InnoCyte Flow Cytometric Cytochrome *c* Release Kit (Calbiochem) according to the manufacturer's instructions.

### Immunofluorescent microscopy

Direct immunofluorescent staining for tubulin was performed as previously described (Verma *et al*, 2008) using anti-*α*-tubulin-FITC conjugate and Hoechst. Images were visualised using a Ziess LSM 510 META imaging system (Carl Ziess MicroImaging, Thornwood, NY, USA).

### Western blot analysis

Whole-cell lysates were obtained using RIPA buffer (Santa Cruz Biotechnology, Santa Cruz, CA, USA), and protein concentration was determined using a NanoDropND-1000 UV-Vis Spectrophotometer (NanoDrop Technologies, Wilmington, DE, USA). Western blot analysis was performed using antibodies directed against BubR1 (Sigma-Aldrich), caspase-8, DR5, Bid, and Bim (Cell Signalling Technology, Danvers, MA, USA), Bcl-2 and Mcl-1 (Calbiochem), and appropriate HRP-conjugated secondary antibody (Dako, Glostrup, Denmark). All blots were reprobed with anti-*β*-actin to confirm equal loading, and densitometry was performed using ImageJ software (US National Institutes of Health; http://rsb.info.nih.gov/ij/).

### TaqMan gene expression analysis

RNA was extracted using the RNeasy Mini Kit (Qiagen, West Sussex, UK), and converted to cDNA using a High Capacity cDNA Reverse Transcription Kit (Applied Biosystems Inc. (ABI), Foster City, CA, USA). TaqMan Gene Expression Assays (DR5, Bim, GAPDH; ABI) and apoptosis panel TaqMan Low Density Arrays (ABI) were performed according to the manufacturer's protocols. Data analysis was performed using the SDSv2.1 program (ABI).

### Statistical analysis

Two-tailed *t*-test analysis was performed using GraphPad InStat v3.05 (GraphPad Software, San Diego, CA, USA), with *P*<0.05 considered significant. PBOX-15-mediated potentiation of TRAIL-induced apoptosis was determined by showing that apoptosis induced by combination treatment was significantly greater than additive (i.e., apoptosis resulting from cotreatment with TRAIL and PBOX-15 was significantly greater than the sum of apoptosis induced by TRAIL alone plus apoptosis induced by PBOX-15 alone).

## Results

### PBOX-15 exhibits anti-myeloma activity *in vitro* and *ex vivo*

**Figure 1:**
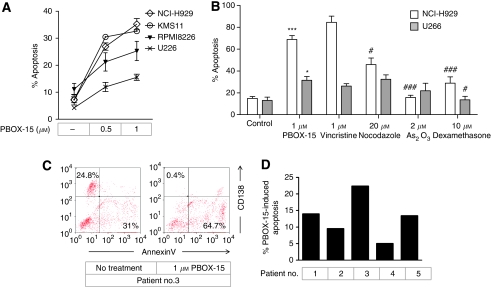
Pyrrolo-1,5-benzoxazepine-15 induces apoptosis in multiple myeloma cells. (**A**) ⋄ NCI-H929, ○ KMS11, ▾ RPMI8226, and **×** U266 cells were treated as indicated for 24 h, and apoptosis was quantified by AnnexinV/PI assay. Data points, mean; bars, s.e.; *n*=3. (**B**) 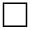 NCI-H929 and 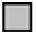 U266 cells were treated as indicated for 24 h, and apoptosis was quantified by AnnexinV/PI assay. Columns, mean; bars, s.e.; *n*=3. ^***^P<0.001, compared with control; ^*^P<0.05, compared with control; ^###^*P*<0.001, compared with PBOX-15; ^#^*P*<0.05, compared with PBOX-15; *t*-test, *n*=3. (**C**) Representative flow cytometry analysis demonstrating concurrent loss of CD138+ expression (upper left quadrant) and increased AnnexinV staining (lower right quadrant) in primary myeloma cells treated with 1 *μ*M PBOX-15 for 24 h. (**D**) Primary multiple myeloma cells were treated with 1 *μ*M PBOX-15 for 24 h and increase in apoptosis, compared with background levels, was quantified by AnnexinV/Hoechst assay.

PBOX-15 was found to induce apoptosis in a dose-dependent manner in a panel of myeloma cell lines, NCI-H929, KMS11, RPMI8226, and U266, although with varying potency. Following treatment with 1 *μ*M PBOX-15 for 24 h, apoptotic responses of 35.2±2.1, 32.7±0.6, and 25.3±3.6% were measured in NCI-H929, KMS11, and RPMI8226 cells, respectively, whereas a lower level of apoptosis, 13.7±2.0%, was measured in U266 cells (Figure 1A). We have previously shown this concentration and duration of exposure to PBOX-15 to be minimally toxic to normal B lymphocytes and bone marrow progenitor cells (McElligott *et al*, 2009). The efficacy of PBOX-15 was next compared with a panel of cytotoxic agents using the cell lines that displayed the least (U266) and most (NCI-H929) sensitivity to PBOX-15. In NCI-H929 cells, PBOX-15-induced apoptosis was found to be comparable to apoptosis induced by 1 *μ*M vincristine (42.3±2.8% *P*>0.05), and greater than apoptosis induced by 10 *μ*M dexamethasone (14.4±2.9% *P*<0.001) or 20 *μ*M nocodazole (14.4±2.9% *P*<0.05) (Figure 1B). NCI-H929 cells were found to be resistant to treatment with 2 *μ*M As_2_O_3_. PBOX-15 was found to induce similar levels of apoptosis in U266 cells as 1 *μ*M vincristine (13.1±1.1% *P*>0.05), 20 *μ*M nocodazole (16.2±2% *P*>0.05), and 2 *μ*M As_2_O_3_ (11±3.4% *P*>0.05), whereas these cells were resistant to dexamethasone-induced apoptosis (Figure 1B).

The effect of PBOX-15 on myeloma cells isolated from bone marrow aspirates of five patients was also assessed. Patients no. 1–4 were newly diagnosed and treatment naive, whereas patient no. 5 had relapsed following an allogeneic haematopoietic stem cell transplant (HSCT) and was lenalidomide refractory. Patient no. 4 had a 17p chromosomal deletion, which is associated with poor clinical outcome (Avet-Loiseau, 2007). Loss of CD138, a myeloma cell-specific transmembrane heparin sulphate proteoglycan, represents a marker for determining the induction of apoptosis in myeloma cells following drug treatment (Jourdan *et al*, 1998; Clendening *et al*, 2010). Here, a PBOX-15-induced decrease in CD138^+^ cells was shown to occur concurrently with increased AnnexinV staining (Figure 1C). Quantitation of apoptosis by AnnexinV/Hoechst staining demonstrated that, following treatment with 1 *μ*M PBOX-15 for 24 h, apoptosis was induced in all samples with a mean increase from background levels of 12±2.9% (range 5–22.4%) (Figure 1D). PBOX-15-induced apoptosis was not further increased in samples treated for up to 72 h (data not shown).

**Figure 2:**
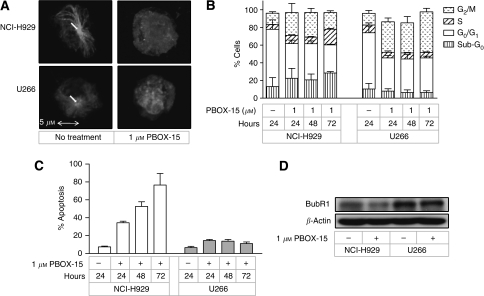
PBOX-15 induces cytoskeleton disruption and G_2_/M arrest in multiple myeloma cell lines, with length of arrest associated with BubR1 expression. (**A**) NCI-H929 and U266 cells were treated as shown for 18 h, after which the tubulin cytoskeleton was visualised by immunofluorescent microscopy using anti-*α*-tubulin-FITC. Microtubules radiating from centrosome are indicated. Results are representative of three independent experiments. (**B**) NCI-H929 and U266 cells were treated as shown, and, following staining with PI, DNA content was analysed by flow cytometry. Columns, mean; bars, s.e.; *n*=3. (**C**) 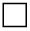 NCI-H929 and 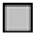 U266 cells were treated with 1 *μ*M PBOX-15 for the times indicated, and apoptosis quantified by AnnexinV/PI assay. Columns, mean; bars, s.e.; *n*=3. (**D**) NCI-H929 and U266 cells were treated as indicated for 24 h, and expression of BubR1 was assessed by western blot analysis. Results are representative of three independent experiments.

Earlier work by our group has shown that the proapoptotic activity of PBOX-15 is associated with the induction of microtubule depolymerisation (Mulligan *et al*, 2006). Here, direct immunofluorescent staining demonstrated that PBOX-15 treatment (1 *μ*M, 18 h) resulted in complete disruption of the microtubule network in both NCI-H929 and U266 myeloma cell lines (Figure 2A). In addition, flow cytometry analysis of PI-stained cells demonstrated that PBOX-15 arrested both cell lines in the G_2_/M phase of the cell cycle (Figure 2B). However, an increase in the sub-G_0_ population, which is indicative of apoptosis, was seen only in PBOX-15-treated NCI-H929 cells. In comparison, G_2_/M arrest was maintained in PBOX-15-treated U266 cells for up to 72 h of treatment. Consistent with this, a time-dependent increase in apoptosis was observed in NCI-H929 cells treated with 1 *μ*M PBOX-15 (Figure 2C), whereas treatment of U266 cells for up to 72 h with 1 *μ*M PBOX-15 did not augment the apoptotic response. Previously, we have shown that cells expressing high levels of the mitotic checkpoint protein BubR1 undergo sustained mitotic arrest in response to treatment with PBOX compounds, whereas a low level of expression is associated with transient arrest and a greater apoptotic response (Greene *et al*, 2008). Similarly, greater expression of BubR1 was detected in U266 cells compared with the NCI-H929 cell line (Figure 2D), and downregulation of BubR1 expression was observed only in NCI-H929 cells after treatment with 1 *μ*M PBOX-15 for 24 h.

### PBOX-15 upregulates DR5 and potentiates TRAIL-induced apoptosis in NCI-H929 and U266 cells

**Figure 3:**
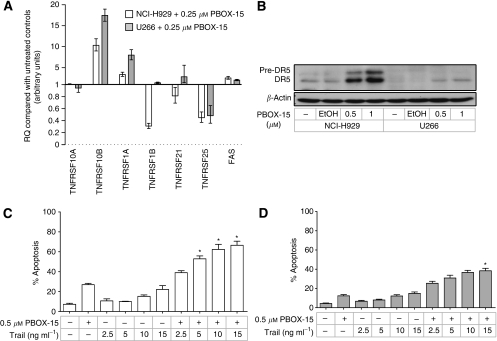
PBOX-15 upregulates DR5 expression and potentiates TRAIL-induced apoptosis in myeloma cell lines. (**A**) 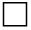 NCI-H929 and 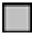 U266 cells were treated with 0.25 *μ*M PBOX-15 for 12 h. Expression of death receptor genes *TNFRSF10A*, *TNFRSF10B*, *TNFRSF1A*, *TNFRSF1B*, *TNFRSF21*, *TNFRSF25*, and *FAS* was analysed using TaqMan LDA apoptosis panels. Relative changes in gene expression (RQ) were calculated compared with untreated controls, using 18S expression as the endogenous control. Columns, mean; bars, RQ_min_ and RQ_max_; *n*=4. (**B**) NCI-H929 and U266 cells were treated as indicated for 24 h and expression of DR5 assessed by western blot analysis. Results are representative of three independent experiments. (**C**) NCI-H929 and (**D**) U266 cells were treated with PBOX-15 and TRAIL as indicated for 24 h, and apoptosis was quantified by AnnexinV/PI assay. Columns, mean; bars, s.e.; *n*=3. Apoptosis due to TRAIL+PBOX-15 was significantly greater than the sum of apoptosis due to TRAIL alone plus apoptosis due to PBOX- 15 alone, ^*^P<0.05, *t*-test, *n*=3.

To delineate the mechanism by which PBOX-15 induces apoptosis, its effect on expression of genes involved in the extrinsic apoptotic pathway was examined in both NCI-H929 and U266 cells. Using preformatted TaqMan Low Density Array apoptosis panels, expression of DR genes *TNFRSF10B*, *TNFRSF1A*, and *FAS*, which encode DR5, tumour necrosis factor receptor-1 (TNF-R1), and Fas, respectively, were found to be upregulated, with relative changes in gene expression (RQ) of >two-fold in both cell lines after treatment with 0.25 *μ*M PBOX-15 for 12 h (Figure 3A). These treatment conditions were used to minimise secondary transcriptional effects due to PBOX-15-induced apoptosis in the cells. The largest fold increase following PBOX-15 treatment of both cell lines was in the expression of *TNFRSF10B* (DR5), with RQ values of 10 and 17 determined for NCI-H929 and U266 cells, respectively. These results were validated using individual TaqMan assays, with similar RQ values calculated (data not shown), and western blot analysis demonstrated upregulation of DR5 protein in both cell lines following treatment with PBOX-15 (Figure 3B). Greater upregulation of DR5 protein was observed in NCI-H929 cells (4.8-fold) following treatment with 1 *μ*M PBOX-15 for 24 h compared with U266 cells (2.9-fold). In addition, upregulation of the DR5 precursor protein was also detected in PBOX-15-treated NCI-H929 cells. DR5 upregulation has previously been associated with enhancement of TRAIL-induced apoptosis in myeloma cells (Jazirehi *et al*, 2001; Mitsiades *et al*, 2001; Liu *et al*, 2003). Cotreatment of NCI-H929 and U266 cells in this study with suboptimal doses of PBOX-15 (0.5 *μ*M) and TRAIL (15 ng ml^−1^) potentiated apoptosis to levels greater than those induced by either agent alone or by their additive effect. In NCI-H929 cells, an apoptotic response of 66.5±4.1% (*P*<0.05) was measured following treatment with 0.5 *μ*M PBOX-15 and 15 ng ml^−1^ TRAIL for 24 h (Figure 3C), and in U266 cells this combination treatment induced 38.4±2.6% (*P*<0.05) apoptosis (Figure 3D).

### PBOX-15-induced apoptosis of NCI-H929 cells is caspase dependent with independent activation of extrinsic and intrinsic apoptotic pathways

**Figure 4:**
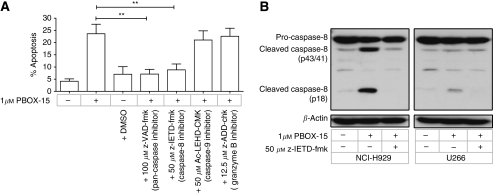
PBOX-15-induced apoptosis in NCI-H929 cells is caspase-8 dependent. (**A**) NCI-H929 cells were treated as indicated for 24 h and apoptosis was assessed by AnnexinV/PI assay. Where indicated, cells were pretreated for 1 h with caspase inhibitor. Columns, mean; bars, s.e.; *n*=4. The pan-caspase inhibitor z-VAD-fmk and the caspase-8 inhibitor z-IETD-fmk significantly inhibited PBOX-15-induced apoptosis, ^**^P<0.01, *t*-test, *n*=4. (**B**) NCI-H929 and U266 cells were treated as indicated for 18 h, and expression of caspase-8 was assessed by western blot analysis. The presence of cleaved p43/p41 and p18 caspase-8 fragments indicates activation of caspase-8 in PBOX-15-treated cells, and this was prevented in cells pretreated with z-IETD-fmk. Results are representative of three independent experiments.

In addition to sensitising cells to DR-ligand-mediated apoptosis, chemotherapy-induced upregulation and clustering of DRs has been shown to directly activate the caspase-8-dependent extrinsic apoptotic pathway (Huang *et al*, 2001). We have previously reported that PBOX-6-induced apoptosis may involve both caspase-dependent and -independent pathways (Zisterer *et al*, 2000; McGee *et al*, 2001), and have recently demonstrated PBOX-15-induced apoptosis of CLL cells to be caspase-8 dependent (McElligott *et al*, 2009). In NCI-H929 cells, pretreatment with the pan-caspase inhibitor z-VAD-fmk completely prevented PBOX-15-induced apoptosis, indicating a caspase-dependent mechanism of apoptosis (Figure 4A). Specifically, PBOX-15-induced apoptosis was found to be caspase-8 dependent, with apoptosis significantly reduced in cells pretreated with the caspase-8 inhibitor z-IETD-fmk (*P*<0.01) (Figure 4A). In comparison, pretreatment of cells with the caspase-9 inhibitor Ac-LEHD-CMK, or the granzyme B inhibitor z-AAD-CMK, did not prevent PBOX-15-induced apoptosis. The activation of caspase-8 during PBOX-15-induced apoptosis of NCI-H929 cells was confirmed by western blot analysis, with cleaved caspase-8 fragments detected after treatment for 18 h with 1 *μ*M PBOX-15 (Figure 4B). In contrast, caspase-8 was cleaved to a lesser extent in PBOX-15-treated U266 cells.

**Figure 5:**
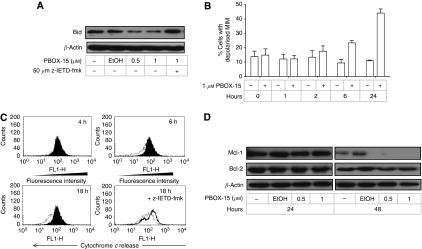
PBOX-15 activates the intrinsic apoptotic pathway in NCI-H929 cells. (**A**) NCI-H929 cells were treated as indicated for 24 h, and expression of Bid was assessed by western blot analysis. Where indicated, cells were pretreated for 1 h with caspase-8 inhibitor z-IETD-fmk. Results are representative of three independent experiments. (**B**) NCI-H929 cells were treated as indicated, and stained with JC-1 for 15 min. Depolarisation of the mitochondrial inner membrane (MIM) was assessed by flow cytometry. Columns, mean; bars, s.e.; *n*=3. (**C**) NCI-H929 cells were treated for the times indicated with 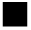 no treatment, --- 1 *μ*M PBOX-15, or **—** 50 *μ*M z-IETD-fmk+1 *μ*M PBOX-15, and assayed for cytochrome *c* release from the mitochondria by flow cytometry using an anti-cytochrome *c* antibody. Reduction in fluorescence is due to release of cytochrome *c* from the mitochondria, followed by its loss through permeabilised cellular membranes. Results are representative of three independent experiments. (**D**) NCI-H929 cells were treated as indicated, and expression of Bcl-2 and Mcl-1 was assessed by western blot analysis. Results are representative of three independent experiments.

In addition to its role in the extrinsic apoptotic pathway, caspase-8 may also trigger the intrinsic apoptotic pathway through cleavage of the proapoptotic protein Bid. Here, we show that expression of Bid was decreased in NCI-H929 cells treated for 24 h with 1 *μ*M PBOX-15, and this was prevented in cells pretreated with z-IETD-fmk (Figure 5A). In support of the activation of the intrinsic apoptotic pathway, flow cytometry analysis of JC-1-stained cells demonstrated that MIM depolarisation occurred in a time-dependent manner in PBOX-15-treated NCI-H929 cells, and was an early event, occurring after 2 h of treatment (Figure 5B). Flow cytometry analysis also showed release of cytochrome *c* from the mitochondria of these cells after 6 h of treatment with PBOX-15 (Figure 5C). However, inhibition of caspase-8 did not prevent MIM depolarisation (data not shown) and only partially prevented cytochrome *c* release (Figure 5C).

### PBOX-15 induces downregulation of Bim_EL_ in NCI-H929 cells

As important regulators of the intrinsic apoptotic pathway, the role of the antiapoptotic Bcl-2 proteins, Bcl-2 and Mcl-1, in PBOX-15-induced apoptosis of NCI-H929 cells was next investigated. No effect on Bcl-2 was observed in NCI-H929 cells following treatment with PBOX-15 for up to 48 h (Figure 5D). However, expression of Mcl-1, which has been shown to be an important regulator of myeloma cell survival (Gomez-Bougie *et al*, 2004), was found to be decreased in NCI-H929 cells treated with 1 *μ*M PBOX-15, with expression completely abolished after 48 h (Figure 5D).

**Figure 6:**
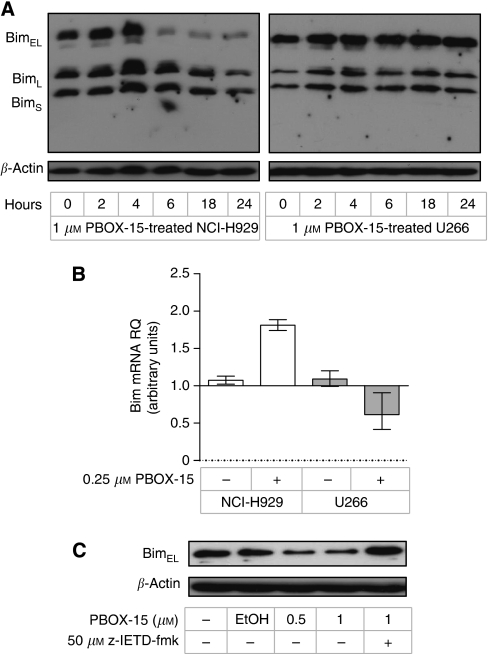
PBOX-15 downregulates Bim in NCI-H929 cells. (**A**) NCI-H929 and U266 cells were treated as indicated, and expression of Bim was assessed by western blot analysis. Results are representative of three independent experiments. (**B**) NCI-H929 and U266 cells were treated as indicated for 12 h, and Bim mRNA levels were assessed by RT–PCR. Relative changes in gene expression (RQ) were calculated compared with untreated controls, using GADPH expression as the endogenous control. Columns, mean; bars, RQ_min_ and RQ_max_; *n*=3. (**C**) NCI-H929 cells were treated as shown for 18 h, and expression of Bim was assessed by western blot analysis. Where indicated, cells were pretreated for 1 h with the caspase-8 inhibitor z-IETD-fmk. Results are representative of three independent experiments.

In myeloma cells, Mcl-1-mediated regulation of apoptosis has been linked to its interaction with the proapoptotic BH3-only protein Bim (Gomez-Bougie *et al*, 2004, 2005). There are three alternative splicing isoforms of Bim (Bim_EL_, Bim_L_, and Bim_S_), and all were found to be expressed in both NCI-H929 and U266 cells (Figure 6A). In NCI-H929 cells, Bim_EL_ expression was found to be decreased after treatment with PBOX-15 for 6 h, with reduced expression maintained for up to 24 h of treatment. A decrease in expression of Bim_L_ and Bim_S_ was also detected in PBOX-15-treated NCI-H929 cells; however, this occurred to a lesser extent, and was not detected until 24 h of treatment. In contrast, PBOX-15 treatment had minimal effect on the expression of any Bim isoform in U266 cells (Figure 6A). RQ–PCR analysis showed an increase in Bim mRNA levels in NCI-H929 cells after treatment with PBOX-15 (Figure 6B), indicating that the observed decrease in Bim_EL_ protein levels is not due to inhibition of gene transcription. In contrast, a decrease in Bim mRNA levels was noted in PBOX-15-treated U266 cells.

Decreased Bim_EL_ protein expression has previously been reported to be the result of caspase-dependent cleavage in myeloma cells undergoing melphalan-induced apoptosis (Gomez-Bougie *et al*, 2005). Here, pretreatment of NCI-H929 cells with the caspase-8 inhibitor, z-IETD-fmk, prevented PBOX-15-induced decrease in Bim_EL_ expression (Figure 6C), suggesting a role for active caspase-8 in downregulating Bim_EL_ expression in these cells.

## Discussion

PBOX-15 is a potent member of a potential new class of anticancer agents that has been shown by us previously to have activity in haematological malignancies, including chemotherapy-refractory CLL and CML cells (McElligott *et al*, 2009; Bright *et al*, 2010). The proapoptotic activity of PBOX-15 has been associated with its tubulin binding and depolymerising properties, and accordingly may be classified as an MTA (Mulligan *et al*, 2006). Microtubule targeting agents are widely used as anti-mitotic agents in the treatment of cancer; however, the mechanism by which they subsequently induce cell death remains incompletely defined and may be heterogeneous both within cell populations and between cell types (Gascoigne and Taylor, 2008). Presently, we investigate the activity of PBOX-15 in myeloma cells, and delineate the mechanism by which it induces apoptosis in these cells. Consequently, we identify a potential new therapeutic approach for the treatment of multiple myeloma.

In this study, the potential of PBOX-15 as a novel anti-myeloma agent was initially demonstrated by its ability to induce apoptosis in a panel of myeloma cell lines. The use of patient samples, however, is important in predicting the clinical relevance of novel anticancer agents, and here we also show PBOX-15-induced apoptosis in *ex vivo* myeloma cells isolated from patient bone marrow aspirates. Of note, we show PBOX-15 activity in myeloma cells isolated from patients with poor clinical prognosis: sample no. 5, which was obtained from a lenalidomide-refractory patient who had relapsed following allogenic HSCT, and sample no. 4, harbouring a 17p deletion associated with loss of p53 expression and aggressive disease (Yeung and Chang, 2008). Previously, we have shown that PBOX-15 induces apoptosis in CLL cells with 17p deletions (McElligott *et al*, 2009). In this study, p53 status appears to influence PBOX-15-induced apoptosis in myeloma cells, with the lowest relative sensitivity to PBOX-15 displayed by patient sample no. 4 and the U266 and RPMI-8226 cells lines, both of which express mutated p53 (Liu *et al*, 2003). This may suggest that PBOX-15 can activate both p53-dependent and -independent apoptotic pathways in myeloma cells.

Of the cell lines tested, NCI-H929 cells exhibited the greatest apoptotic response following treatment with PBOX-15, whereas the U266 cell line was the least sensitive. However, in both cell lines, PBOX-15-induced apoptosis was found to be comparable to, or greater than, that induced by other cytotoxic agents, including the MTAs vincristine and nocodazole, and the anti-myeloma agent dexamethasone. Despite their differential sensitivities to PBOX-15, both NCI-H929 and U266 cells were found to undergo cytoskeleton disruption and G_2_/M phase cell cycle arrest following treatment with PBOX-15. However, in contrast to the subsequent potent induction of apoptosis in NCI-H929 cells, PBOX-15-treated U266 cells were found to undergo sustained cell cycle arrest. Previously, we have demonstrated the duration of G_2_/M arrest induced by PBOX compounds to correlate with endogenous expression levels of the mitotic spindle checkpoint protein BubR1 (Greene *et al*, 2008). Here, we show greater expression of BubR1 in U266 cells compared with the NCI-H929 cell line, and demonstrate downregulation of BubR1 expression in NCI-H929 cells, but not U266 cells, following treatment with PBOX-15. Thus, differences in BubR1 expression levels may account in part for the different cytostatic responses of NCI-H929 and U266 cells to PBOX-15.

Significantly, we show PBOX-15-induced upregulation of the DR5, TNF-R1, and Fas DR genes in both NCI-H929 and U266 cells, suggesting that PBOX-15 may sensitise myeloma cells to DR-mediated apoptosis. In both cell lines, the greatest increase in gene expression was of *TNFRSF10B*, with a corresponding upregulation of the gene product, DR5. Importantly, the level of PBOX-15-induced DR5 upregulation was found to correlate with the subsequent level of apoptosis induced in the two cell lines. Together, these data suggest a primary role for the extrinsic apoptotic pathway in PBOX-15-induced apoptosis of myeloma cells. Consistent with this, we found PBOX-15-induced apoptosis in NCI-H929 cells to be caspase-8 dependent. Microtubule targeting agent-induced upregulation of DR5 has previously been shown to sensitise breast and ovarian cancer cells to TRAIL-induced apoptosis (LaVallee *et al*, 2003; Wood *et al*, 2004). Similarly, upregulation of DR5 has been associated with chemosensitisation of myeloma cells to TRAIL-induced apoptosis following treatment with doxorubicin, As_2_O_3_, the histone deacetylase inhibitor Trichostatin A, or the Akt inhibitor perifosine (Jazirehi *et al*, 2001; Liu *et al*, 2003; Fandy and Srivastava, 2006; David *et al*, 2008). However, to the best of our knowledge, MTA-induced chemosensitisation of myeloma cells to TRAIL through DR5 upregulation has not previously been reported. Here, we show that, consistent with DR5 upregulation, cotreatment of both NCI-H929 and U266 cells with suboptimal doses of PBOX-15 and TRAIL potentiated the apoptotic response. Thus, in addition to its potential as a single agent, these results identify a potential role for PBOX-15 as a novel chemosensitiser in the treatment of myeloma. Of particular note is the ability of PBOX-15 to sensitise the relatively chemoresistant U266 cell line to TRAIL-induced apoptosis. Thus, together with our demonstration of PBOX-15-induced apoptosis in lenalidomide-refractory patient sample no. 4, and our previous work identifying the ability of PBOX-15 to induce apoptosis in p-glycoprotein-positive and breast cancer resistance protein-positive cancer cells (Nathwani *et al*, 2009), both of which have been shown to be associated with chemotherapy-induced treatment drug resistance in myeloma (Grogan *et al*, 1993; Turner *et al*, 2006), we suggest a potential role for PBOX-15 in the treatment of chemoresistant myeloma.

Crosstalk between the extrinsic apoptotic pathway and the intrinsic pathway, the other main mechanism by which apoptosis can proceed, was suggested by the detection of a caspase-8-dependent decrease in Bid expression in PBOX-15-treated NCI-H929 cells. However, although inhibition of caspase-8 partially prevented mitochondrial cytochrome *c* release in these cells, MIM depolarisation was caspase-8 independent. Moreover, pharmacological inhibition of caspase-9, a key mediator of the intrinsic pathway, did not prevent PBOX-15-induced apoptosis. Together, these results demonstrate that the intrinsic and extrinsic apoptotic pathways are independently triggered in PBOX-15-treated NCI-H929 cells. These data also identify the extrinsic pathway as the primary mechanism by which PBOX-15-induced apoptosis proceeds in NCI-H929 cells, and suggests that the role of the intrinsic apoptotic pathway here is in amplification, rather than direct initiation, of apoptosis.

We have previously reported a role for antiapoptotic Bcl-2, an important regulator of the intrinsic apoptotic pathway, in PBOX-induced apoptosis of CML cells (Mc Gee *et al*, 2004). Although no role for Bcl-2 in PBOX-15-induced apoptosis of NCI-H929 cells was observed in this study, antiapoptotic Mcl-1 was found to be downregulated in these cells. In myeloma cells, Mcl-1-mediated apoptosis has been reported to be regulated by its interaction with proapoptotic Bim (Gomez-Bougie *et al*, 2004, 2005; Morales *et al*, 2008). Here, a decrease in expression of Bim was detected in NCI-H929 cells treated with PBOX-15, with downregulation of the Bim_EL_ isoform observed to be an early event. As a proapoptotic protein, downregulation of Bim is more usually associated with cell survival and antiapoptotic signals (Craxton *et al*, 2005; Morales *et al*, 2008), whereas increased expression is associated with apoptosis of myeloma cells (Gómez-Benito *et al*, 2007; Morales *et al*, 2008). However, the apoptotic activity of Bim may also be regulated by other mechanisms such as post-translational modification (Lomonosova and Chinnadurai, 2009). Here, decreased Bim expression in PBOX-15-treated NCI-H929 cells was shown not to result from transcriptional repression, but instead was found to be caspase-8 dependent. Decreased expression of Bim_EL_ has previously been described to result from caspase-dependent generation of N-terminally cleaved Bim_EL_, which displays increased proapoptotic activity, during apoptotic signalling in the Jurkat T-lymphocytic leukaemia cell line (Chen and Zhou, 2004). A comparable role for cleaved Bim_EL_ in amplification of apoptosis has also been suggested in melphalan-treated myeloma cells (Gomez-Bougie *et al*, 2005). However, the role of caspase-8-dependent downregulation of Bim_EL_ during PBOX-15-induced apoptosis of NCI-H929 cells is unclear, and a potential role for cleaved Bim in the amplification of PBOX-15-induced apoptosis in myeloma cells warrants further investigation.

Overall, this study demonstrates the potential of PBOX-15 as an anti-myeloma agent, and we show activation of multiple apoptotic mechanisms in PBOX-15-treated myeloma cells. Functional studies have identified upregulation of DR5 and activation of caspase-8 as key mechanisms by which PBOX-15 induces apoptosis in myeloma cells. In addition, the ability of PBOX-15 and TRAIL to potentiate apoptosis of myeloma cells through DR5 upregulation identifies a novel mechanism underlying the potential use of PBOX-15 as a strategy for chemosensitisation of myeloma cells. Additional preclinical studies, including the use of animal models, are warranted to further assess the potential of PBOX-15 as a potential therapeutic agent for myeloma.
